# Role of Acetic Acid and Nitric Oxide against Salinity and Lithium Stress in Canola (*Brassica napus* L.)

**DOI:** 10.3390/plants13010051

**Published:** 2023-12-22

**Authors:** Mona F. A. Dawood, Md. Tahjib-Ul-Arif, Abdullah Al Mamun Sohag, Arafat Abdel Hamed Abdel Latef

**Affiliations:** 1 Botany and Microbiology Department, Faculty of Science, Assiut University, Assiut 71516, Egypt; mo_fa87@aun.edu.eg; 2 Department of Biochemistry and Molecular Biology, Faculty of Agriculture, Bangladesh Agricultural University, Mymensingh 2202, Bangladesh; sohag2010bmb.sust@gmail.com; 3 Botany and Microbiology Department, Faculty of Science, South Valley University, Qena 83523, Egypt

**Keywords:** antioxidant system, exogenous chemicals, combined stress, heavy metal stress, plant growth, salt stress

## Abstract

In this study, canola (*Brassica napus* L.) seedlings were treated with individual and combined salinity and lithium (Li) stress, with and without acetic acid (AA) or nitric acid (NO), to investigate their possible roles against these stresses. Salinity intensified Li-induced damage, and the principal component analysis revealed that this was primarily driven by increased oxidative stress, deregulation of sodium and potassium accumulation, and an imbalance in tissue water content. However, pretreatment with AA and NO prompted growth, re-established sodium and potassium homeostasis, and enhanced the defense system against oxidative and nitrosative damage by triggering the antioxidant capacity. Combined stress negatively impacted phenylalanine ammonia lyase activity, affecting flavonoids, carotenoids, and anthocyanin levels, which were then restored in canola plants primed with AA and NO. Additionally, AA and NO helped to maintain osmotic balance by increasing trehalose and proline levels and upregulating signaling molecules such as hydrogen sulfide, γ-aminobutyric acid, and salicylic acid. Both AA and NO improved Li detoxification by increasing phytochelatins and metallothioneins, and reducing glutathione contents. Comparatively, AA exerted more effective protection against the detrimental effects of combined stress than NO. Our findings offer novel perspectives on the impacts of combining salt and Li stress.

## 1. Introduction

Salinity-induced decrements to plant growth and yield are ongoing problems due to poor cultivation practices and climate change, impacting nearly 1125 million hectares of agricultural land [1]. Several regions, such as the Middle East, North Africa, Central Asia, the Pacific, and South America and are particularly affected by salinity [2]. Most previous studies have been conducted to reduce salinity stress or the single stressors affecting crops. However, in field conditions, numerous abiotic stresses can coexist rather than a single stress. This simultaneous stress can be highly detrimental to crops, yet researchers have barely studied its effects and potential solutions [3]. Some recent studies on simultaneous stress have revealed the unique responses of different plants to combinations of stressors and the difficulty of extrapolating from responses to individual stressors [4,5,6]. Additionally, with the advancement of technology and the influence of anthropogenic factors, certain emerging contaminants can substantially impact crop productivity. Therefore, it is crucial to simulate combined stress conditions or field environments in future research to enhance crops’ acclimatization to both natural and anthropogenic stresses.

Lithium (Li) is the 27th most abundant element, is highly reactive, and exists as salts or minerals. Lithium is eluted to the ecosystem in the form of industrial wastewater contaminated with Li, discarded batteries containing Li, the use of industrial colorants rich in Li, and Li brines. In aqueous environments, the dissolved forms of Li compounds have high solubility and are relatively inert [7]. However, most plants can absorb Li from their growing environment, and its threshold levels vary significantly and can cause moderate to severe detrimental effects at 4–40 mg Li kg^−1^ [8,9]. Lithium may have the same routes and transport passages across cell membranes as K^+^ and, therefore, can be efficiently absorbed and moved to leaves, competing with Ca^2+^ in the roots of some plants. Consequently, Li-contaminated soils could significantly affect Ca^2+^ uptake and transport in plants [9,10].

Thus, the excessive presence of Li and salt can impact plants by stimulating the generation of reactive oxidative species (ROS), which are harmful to plant cells. ROS can cause lipid peroxidation, protein oxidation, and inactivation of numerous important enzymes [11], ultimately affecting plant growth, development [12], the photosynthetic system [13], ionic homeostasis [14,15], and secondary metabolite metabolism [16]. In response to these detrimental consequences, plants employ several strategies to acclimatize, including morphological adaptation [17], synthesis of osmoprotectants [15], and utilization of secondary metabolites and enzymatic and nonenzymatic mechanisms [18]. Among these mechanisms, a well-balanced antioxidant system plays a decisive function in maintaining overall plant health, helping to detoxify excessive ROS produced under salt and Li stress [18].

In recent years, the application of exogenous signaling molecules has gained significant attention for improving stress tolerance due to their simplicity and quick responses. Nitric oxide (NO) and acetic acid (AA) are two widely studied protective compounds in plant stress physiology. They have attracted attention for their potential role in reducing plant stresses [19,20,21,22]. Acetic acid, an important metabolite in plants, has a significant role in promoting stress tolerance and is associated with gene expression regulation and epigenetic activation [23]. Rasheed et al. [24] found that AA application could be utilized as an energy source to preserve cellular energy in plants under abiotic/biotic stresses. In this regard, Cronan and Laporte [25] found that AA acts instantaneously as a substrate for acetyl-CoA synthetase, producing acetyl-CoA that is incorporated into the tricarboxylic acid and glyoxylate cycles. AA has been used to meliorate the damaging impacts of salinity [19,26] and drought [27], but its utilization for combined salinity and Li-stress has not been investigated yet.

NO modulates growth characteristics and multifaceted upregulatory mechanisms from seed germination to root development, and as well as leaves’ stomatal movement and maturation, thereby providing plant protection. Furthermore, NO has been found to function against various stressors by mediating oxidative stress, antioxidant responses, ion re-establishment, and metal transport, and it also upregulates the activities of some transcription factors in plants [28]. Its potential against multiple stressors, such as heavy metals, salinity, temperature, and waterlogging, has been extensively documented [21,29,30,31]. However, its regulatory role against combined stressors, such as salinity and lithium, remains to be discovered.

Canola, or rapeseed (*Brassica napus* L.), a member of the Brassicaceae family, is cultivated worldwide for edible oil and biodiesel fuel. Moreover, it ranks as the third most important oil crop, following palm and soybean. While some varieties are susceptible to salt stress, which significantly impacts yield and production [32,33], others have been reported to be susceptible to heavy metal stress [34,35]. However, the effects of combined salinity and Li stress remain unclear. In addition, the comparative roles of AA and NO in reducing individual and combined salinity and Li stress have not been examined yet. Therefore, the present investigation aims to evaluate the impact of individual and combined salt and Li stress on canola plants and explore the potential of NO or AA priming under these conditions. To achieve these purposes, various morphophysiological and biochemical approaches were employed. We assessed (1) morphological parameters such as lengths of shoot (SL) and root (RL), plant fresh weight (PFW), plant dry weight (PDW), degree of root browning (DRB), and relative water content (RWC); (2) reactive oxygen species (ROS) and membrane damaging criteria (O_2_**^•^**^−^, ^1^O_2_, ^•^OH, H_2_O_2,_ malondialdehyde, lipoxygenase, electrolyte leakage, and MG); (3) osmolytes (trehalose and proline) and ions (sodium and potassium); (4) enzymatic antioxidants (SOD, CAT, POX, APX, GPX, and PPO); (5) nonenzymatic antioxidants and secondary metabolites (ASA, GSH, TOC, ANC, TPC, FLA, and Car); and other important stress regulators, such as glutathione-S-transferase (GST), phytochelatins (PC), phenylalanine ammonia lyase (PAL), metallothioneins (MT), NO, hydrogen sulfide (H_2_S), cysteine (Cys), salicylic acid (SA), and γ-aminobutyric acid (GABA). This study is expected to provide valuable information regarding the role of AA or NO in inducing resilience against combined salinity and lithium stress, while also figuring out the underlying mechanisms of stress impact in canola plants.

## 2. Results

### 2.1. Effects of Exogenous AA or NO on Growth, Photosynthetic Pigments, and Water Relations

**Figure 1:**
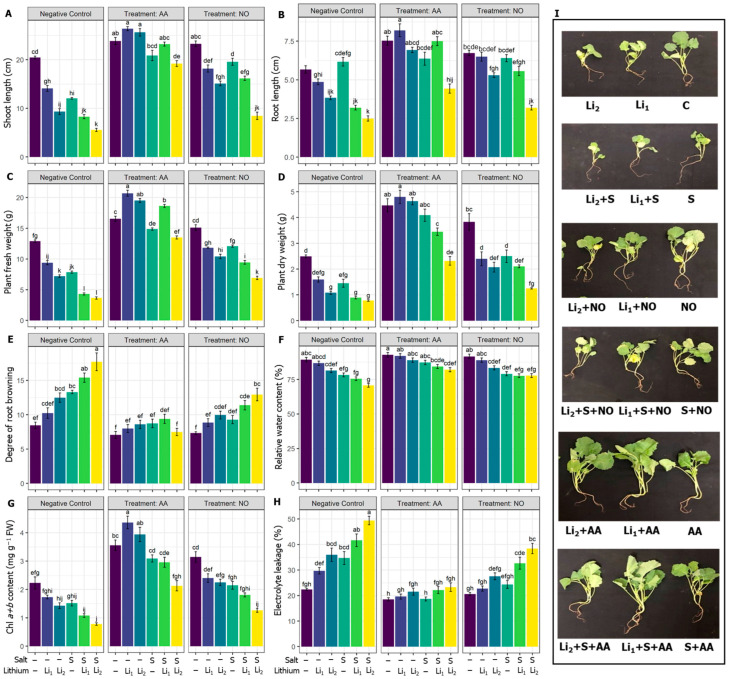
Effects of exogenous acetic acid (AA) or nitric oxide (NO) application on (**A**) shoot length, (**B**) root length, (**C**) plant fresh weight, (**D**) plant dry weight, (**E**) degree of root browning, (**F**) relative water content, (**G**) Chl *a*+*b* content, (**H**) electrolyte leakage, and (**I**) phenotypic appearance of canola seedlings grown under the presence or absence of different concentrations of lithium (Li) and salinity (S) stress. ‘C’—control (non-treated soil); ‘Li_1_’— 50 mg Li_2_CO_3_ kg^−1^ soil; ‘Li_2_’—100 mg Li_2_CO_3_ kg^−1^ soil; ‘S’—100 mM NaCl; ‘AA’—16 mM acetic acid; and ‘NO’—100 μM nitric oxide. Vertical bar diagrams represent the means of three independent replicates (n = 5). Vertical lines at the top of the bars indicate standard errors. Different letters in a graph (where three panels—‘Negative Control’, ‘Treatment: AA’, and ‘Treatment: NO’—together are considered as one graph) represent significant differences based on Tukey’s test at a 5% level of probability (*p* < 0.05).

Compared with the untreated control condition, SL in the canola plant decreased significantly in all the stress treatments, and a drastic reduction was noticed in the combined stress (S+Li_1_ and S+Li_2_) treatments. However, the exogenous AA or NO application improved the SL of canola plants in all treatments compared to their negative control conditions, except the NO+S+Li_2_ treatment (Figure 1A). In comparison with the untreated control, the RL in canola plants reduced significantly in all the treatments except Li_1_ and S conditions; however, the exogenous AA or NO application augmented the RL of canola plants in most treatments when compared with their respective negative control conditions, except AA+S, NO+S and NO+S+Li_2_ (Figure 1B). The PFW and PDW decreased in all the treatments compared with the untreated control condition, where the most significant reduction was noticed under combined stress conditions. However, AA or NO significantly improved the PFW of canola plants, with AA showing the most significant improvement when compared with their respective negative control treatments (Figure 1C). AA or NO pretreatment significantly improved the PDW of canola plants in all the treatments compared to their respective negative control treatments, except NO+Li_1_ and NO+S+Li_2_ (Figure 1D). Single or combined stressors intensified the DRB, whichever stressor was applied, except for Li_1_ compared to the untreated control treatment. However, AA priming significantly decreased the DRB in all the treatments except AA+Li_1,_ when compared with their respective stress conditions. NO priming significantly decreased the DRB in all the treatments except NO+Li_1_ and NO+Li_2,_ when compared with their respective stress conditions (Figure 1E). In comparison with the untreated control, RWC in canola plants significantly decreased in S, Li_1_+S and Li_2_+S treatments. However, the exogenous application of AA significantly improved RWC in those treatments, while exogenous NO did not alter RWC, whichever the treatment, when compared with their respective stress conditions (Figure 1F).

Regarding the photosynthetic pigments, Chl *a+b* content significantly decreased in canola plants under saline and/or Li levels compared to unstressed plants except for Li_1_ and S treatments. However, exogenous AA or NO increased the Chl *a+b* content in all treatments compared to their corresponding stress conditions, except for the NO+Li_2_ treatment (Figure 1G). Electrolyte leakage significantly increased in all treatments except Li_1_ compared to the untreated control condition. On the other hand, exogenous AA decreased the electrolyte leakage in all the treatments compared to their respective stress conditions. Exogenous NO also showed a significant reduction in the electrolyte leakage values of the stressed plants, except NO+Li_2,_ when compared with their respective stress conditions (Figure 1H).

### 2.2. Effects of AA or NO on ROS Contents and Membrane Protection

**Figure 2:**
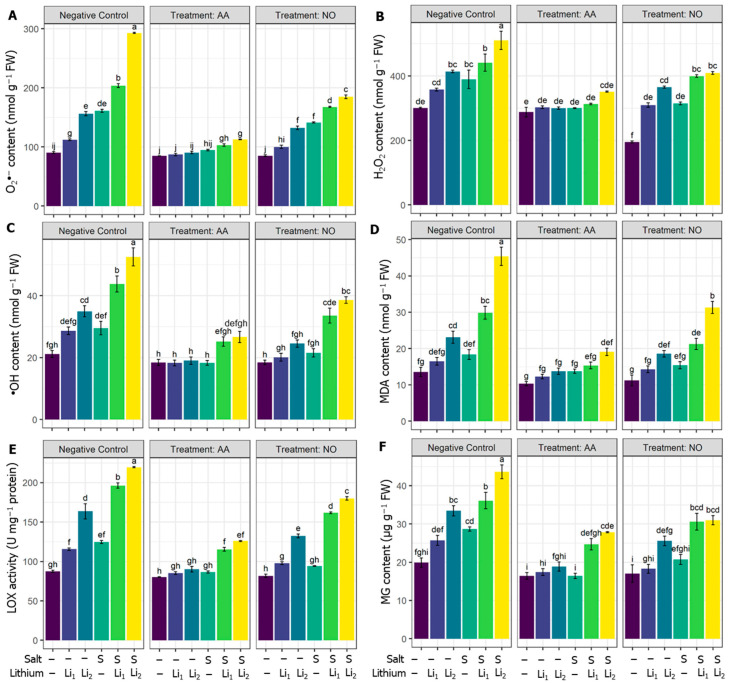
Effects of exogenous acetic acid (AA) or nitric oxide (NO) application on (**A**) superoxide content, (**B**) hydrogen peroxide content, (**C**) hydroxy radical content, (**D**) malondialdehyde content, (**E**) lipoxygenase activity, and (**F**) methyl glyoxal content of canola seedlings grown under the absence or presence of different concentrations of lithium (Li) and/or salinity (S) stress. ‘C’—control (nontreated soil); ‘Li_1_’—50 mg Li_2_CO_3_ kg^−1^ soil; ‘Li_2_’—100 mg Li_2_CO_3_ kg^−1^ soil; ‘S’—100 mM NaCl; ‘AA’—16 mM acetic acid; and ‘NO’—100 μM nitric oxide. Vertical bar diagrams represent the means of three independent replicates (n = 5). Vertical lines at the top of the bars indicate standard errors. Different letters in a graph (where three panels—‘Negative Control’, ‘Treatment: AA’, and ‘Treatment: NO’—together are considered as one graph) represent significant differences based on Tukey’s test at a 5% level of probability (*p* < 0.05).

Compared with the untreated control condition, O_2_**^•^**^−^ content triggered highly significantly in all the treatments, with a high effect in combined stress. The exogenous AA and NO decreased the O_2_**^•^**^−^ content in all the treatments compared to their respective stress conditions (Figure 2A). H_2_O_2_ content was significantly elevated, whatever the stressor applied, compared to unstressed plants, except Li_1_ treated plants. However, exogenous AA diminished the H_2_O_2_ content in S, Li, Li_2_, and their combinations, except for AA+Li_1,_ when compared with their respective stress conditions. NO application also displayed a significant decrement in H_2_O_2_ content under NO+S and NO+S+Li_2_ treatments compared to the stressed plants (Figure 2B). Also, ^•^OH content was significantly triggered by Li_2_ and combined stressors, while Li_1_ and S treatments recorded nonsignificant increments compared to unstressed plants. AA or NO applications minimized the ^•^OH content for most treatments compared to their respective stress conditions, except for NO+Li_1_ and NO+S (Figure 2C).

The membrane-damaging traits were also analyzed in terms of MDA content, which was nonsignificantly increased under Li_1_ and S treatments, while a highly significant increment was noted for the other treatments. Otherwise, AA priming reduced MDA levels, especially for combined stressors, except for AA+Li_1_ and AA+S when compared to their respective stress conditions. Exogenous NO reduced the MDA level in NO+S+Li_1_ and NO+S+Li_2_ significantly relative to their respective stress conditions (Figure 2D). The activity of LOX in the canola plant also significantly increased in all the treatments except the Li_1_ treatments. However, AA and NO attenuated the activity of LOX in all the treatments compared to their respective stress conditions (Figure 2E). Compared with the untreated control condition, the MG content of canola plants significantly increased under the individual and combined stressors, except Li_1_, while AA and NO application retarded the MG content under most conditions except for NO+S+Li_1_ treatment compared with their respective stress conditions (Figure 2F). 

### 2.3. Effects of AA or NO on Non-Enzymatic Antioxidants and Secondary Metabolites

**Figure 3:**
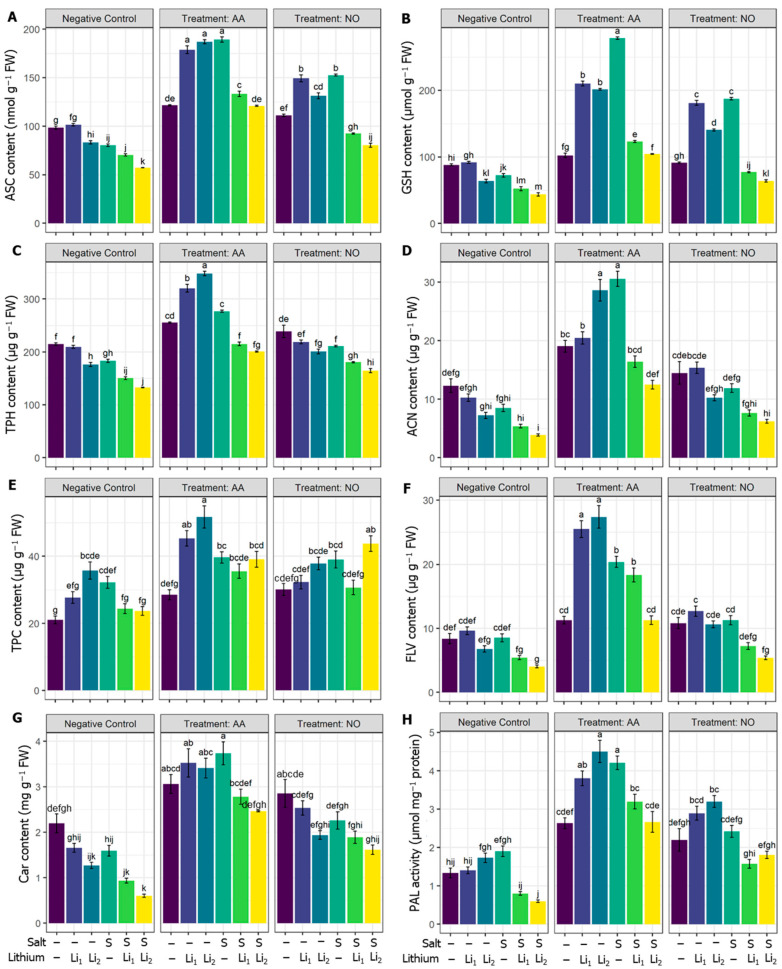
Effects of exogenous acetic acid (AA) and nitric oxide (NO) on (**A**) ascorbic acid (ASC) content, (**B**) glutathione (GSH) content, (**C**) α-Tocopherol (TPH) content, (**D**) anthocyanins (ACN) content, (**E**) total phenolic compounds (TPC) content, (**F**) flavonoids (FLV) content, (**G**) carotenoids (Car) content, and (**H**) phenylalanine ammonia lyase (PAL) activity of canola seedlings grown with or without different concentrations of lithium (Li) and salinity (S) stress. ‘C’—control (non-treated soil); ‘Li_1_’—50 mg Li_2_CO_3_ kg^−1^ soil; ‘Li_2_’—100 mg Li_2_CO_3_ kg^−1^ soil; ‘S’—100 mM NaCl; ‘AA’—16 mM acetic acid; and ‘NO’—100 μM nitric oxide. Vertical bar diagrams represent the means of three independent replicates (n = 5). Vertical lines at the top of the bars indicate standard errors. Different letters in a graph (where three panels—‘Negative Control’, ‘Treatment: AA’, and ‘Treatment: NO’—together are considered as one graph) represent significant differences based on Tukey’s test at a 5% level of probability (*p* < 0.05).

In comparison with the untreated control condition, the ASC, GSH, and TPH contents in canola plants were significantly decreased in stressed plants except Li_1_ (Figure 3A–C). On the other hand, AA and NO application increased the ASC and GSH contents in all the treatments when compared with their respective stress treatments (Figure 3A,B). AA or NO pretreatment enhanced the TPH in most treatments, except for NO+Li_1,_ relative to their corresponding stress conditions (Figure 3C).

The secondary metabolites, such as can, contents were significantly decreased under combined stressors (S+Li_1_ and S+Li_2_). On the other hand, exogenous AA improved the ACN content in stressed and non-stressed plants compared to their respective stress treatment, but NO exerted a nonsignificant effect (Figure 3D). In Figure 3E, the content of TPC was increased under the stress applied, significantly for Li_2_ and S. AA application stimulated the TPC in the AA+Li_1_ and AA+Li_2_ treatments when compared with their respective stress conditions (Figure 3E). The exogenous NO exhibited a significant increase only in NO+S+Li_2_ when compared with its stress condition (Figure 3E). Compared with untreated control plants, FLV significantly decreased under S+Li_2_ treatment. However, using AA highly significantly upregulated the FLV content for stressed and non-stressed plants (Figure 3F) and NO to a lesser extent significantly enhanced the FLV with all stressors except for NO+S+Li_2_ when compared with their respective stress conditions (Figure 3F). Car content was non-significantly reduced by S and Li_1_, but significant reductions were recorded for Li_2_, S+Li_1_ and S+Li_2_. Also, remarkable stimulation of Car content was the result of AA priming whatever the treatment applied (Figure 3G), and exogenous NO significantly increased only in NO+S+Li_1_ and NO+S+Li_2_ when compared with their respective stress conditions (Figure 3G). Compared with untreated control canola plants, PAL activity was significantly decreased in S+Li_1_ and S+Li_2_. However, exogenous AA increased the PAL in all the treatments compared to their respective stress conditions. Exogenous NO also increased the PAL in all the treatments except NO+S compared to their respective stress conditions (Figure 3H).

### 2.4. Effects of AA or NO on Enzymatic Antioxidants

**Figure 4:**
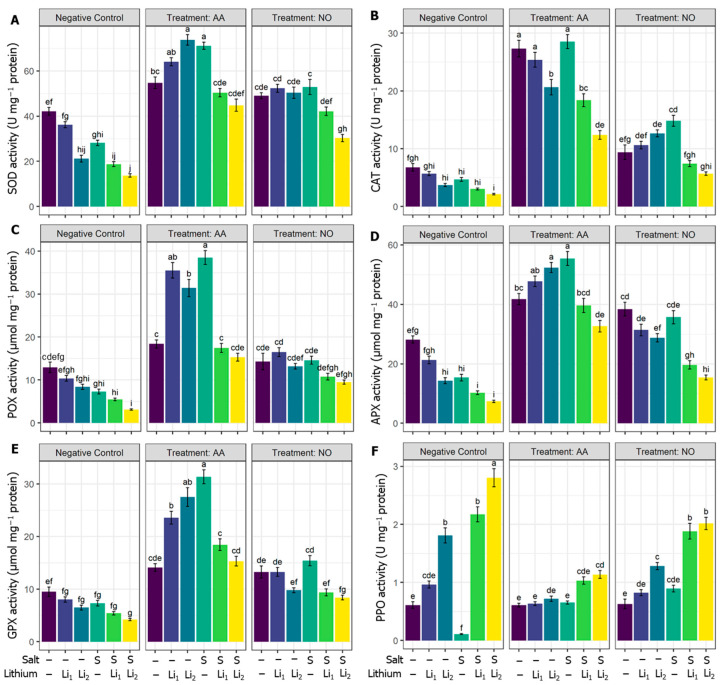
Effects of exogenous acetic acid (AA) and nitric oxide (NO) on (**A**) superoxide dismutase (SOD) activity, (**B**) catalase (CAT) activity, (**C**) peroxidase (POX) activity, (**D**) ascorbate peroxidase (APX) activity, (**E**) glutathione peroxidase (GPX) activity, and (**F**) polyphenol oxidase (PPO) activity of canola seedlings grown under the presence or absence of different concentrations of lithium (Li) and/or salinity (S) stress. ‘C’—control (non-treated soil); ‘Li_1_’—50 mg Li_2_CO_3_ kg^−1^ soil; ‘Li_2_’—100 mg Li_2_CO_3_ kg^−1^ soil; ‘S’—100 mM NaCl; ‘AA’—16 mM acetic acid; and ‘NO’—100 μM nitric oxide. Vertical bar diagrams represent the means of three independent replicates (n = 5). Vertical lines at the top of the bars indicate standard errors. Different letters in a graph (where three panels—‘Negative Control’, ‘Treatment: AA’, and ‘Treatment: NO’—together are considered as one graph) represent significant differences based on Tukey’s test at a 5% level of probability (*p* < 0.05).

The applied stressors adversely downregulated the SOD activity significantly, except for Li_1,_ compared to untreated control canola plants. In contrast, the utilization of AA and NO increased the SOD activity in all the treatments compared to their respective stress conditions (Figure 4A). Concerning untreated control canola plants, CAT activity decreased under S and/or Li stress, and significantly for S+Li_2_. Both protectants alleviated the reduction in CAT under stress conditions, but much more so for AA priming. Exogenous NO non-significantly affects CAT activity under combined stressors (NO+S+Li_1_ and NO+S+Li_2_) when compared with their respective stress conditions (Figure 4B). Concerning POX activity, single stressors non-significantly reduced its activity; while significant reductions were recorded under combined stressors (S+Li_1_ and S+Li_2_) compared with untreated control canola plants. However, APX activity showed high sensitivity to single or combined stressors, except for Li_1,_ while highly significant retardation was denoted for S+Li_2_ compared to untreated control plants.

Both AA and NO vastly upregulated the activities of POX and APX under various conditions, but NO failed to induce a significant increment of POX activity for the combined stressors (NO+Li_2_ and NO+S+Li_2_) when compared with their respective stress conditions (Figure 4C,D). Compared with untreated control canola plants, the activity of GPX significantly decreased in S+Li_2_. Exogenous AA increased the activity of GPX in all the treatments compared with their respective stress conditions. Exogenous NO showed a significant increase in GPX activity in all the treatments, except NO+S+Li_1_ and NO+S+Li_2,_ when compared with their respective stress conditions (Figure 4E). PPO exhibited variable response under stress conditions and exogenous modulator treatments (Figure 4F). Salinity stress highly significantly retarded the activity of PPO, while Li_2_ and combined stressors increased its activity higher than the unstressed control. The applied protectants further reduced the activity of PPO under salinity stress, while they kept the activity of PPO comparable to unstressed plants for single stressors, and reduced the activity of PPO for combined stressors to lower than the corresponding stressed plants only.

### 2.5. Effects of AA or NO on Trehalose and Proline Contents, and Na^+^ and K^+^ Homeostasis

**Figure 5:**
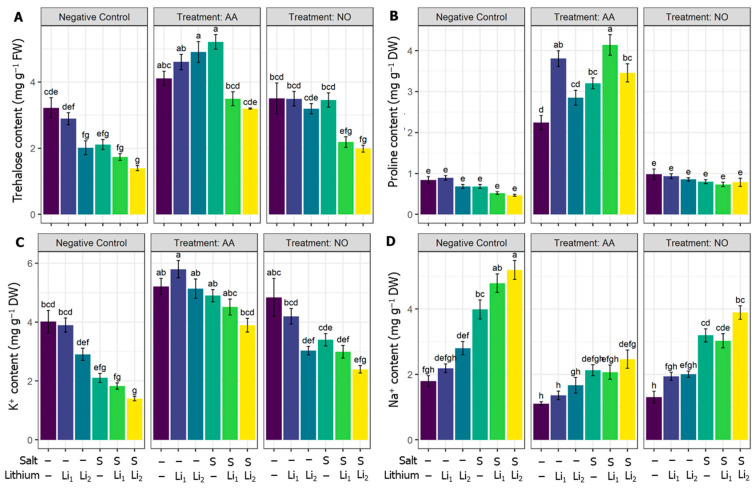
Effects of exogenous acetic acid (AA) and nitric oxide (NO) on (**A**) trehalose content, (**B**) proline content, (**C**) K^+^ content, and (**D**) Na^+^ content of canola seedlings grown with or without different concentrations of lithium (Li) and/or salinity (S) stress. ‘C’—control (non-treated soil); ‘Li_1_’—50 mg Li_2_CO_3_ kg^−1^ soil;—‘Li_2_’—100 mg Li_2_CO_3_ kg^−1^ soil; ‘S’—100 mM NaCl; ‘AA’—16 mM acetic acid; and ‘NO’—100 μM nitric oxide. Vertical bar diagrams represent the means of three independent replicates (n = 5). Vertical lines at the top of the bars indicate standard errors. Different letters in a graph (where three panels—‘Negative Control’, ‘Treatment: AA’, and ‘Treatment: NO’—together are considered as one graph) represent significant differences based on Tukey’s test at a 5% level of probability (*p* < 0.05).

As presented in Figure 5A, trehalose was highly significantly reduced by salinity and Li_2_. However, salinity intensified the rate of trehalose reduction when coapplied with lithium, even at Li_1_ (single Li_1_ does not affect trehalose content), compared to untreated control canola plants. However, AA supplementation increased the trehalose content in all treatments compared to their respective stress conditions. Exogenous NO also showed a significant increase in the NO+Li_2_ and NO+S conditions (Figure 5A).

Although proline content decreased under saline and/or Li stress, this reduction was not significant whatever the stress applied. However, exogenous AA stimulated the production of proline, whatever the stress imposed, compared to their respective stress conditions. Exogenous NO could not significantly alter the proline content compared to their respective treatments (Figure 5B). Compared with untreated control canola plants, K^+^ content significantly decreased in S, S+Li_1_ and S+Li_2_ treatments. However, AA application hindered the reduction in K^+^ under single or combined stressors, relative to the corresponding stress conditions. Exogenous NO showed non-significant increments in K^+^ under various stressors, relative to corresponding stress conditions only (Figure 5C). As represented in Figure 5D, Na^+^ content was enhanced significantly by salinity stress and excessive accumulation was recorded for S+Li_1_ and S+Li_2_ treatments relative to untreated control canola plants. AA significantly attenuated the accumulation of Na^+^ in canola leaves for AA+S, AA+S+Li_1_, and AA+S+Li_2_ conditions relative to their stress levels. While NO exerted a significant reduction in the content of Na for combined stress (NO+S+Li_1_ and NO+S+Li_2_) compared to the stress level only (Figure 5D).

### 2.6. Effects of AA or NO on Li-Chelation-Related Parameters

**Figure 6:**
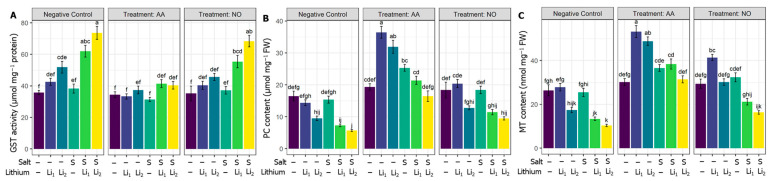
Effects of exogenous acetic acid (AA) or nitric oxide (NO) on (**A**) glutathione-S-transferase (GST) content, (**B**) phytochelatins (PC) content, and (**C**) metallothioneins (MT) content of canola seedlings grown with and without the presence of different concentrations of lithium (Li) and/or salinity (S) stress. ‘C’—control (non-treated soil); ‘Li_1_’—50 mg Li_2_CO_3_ kg^−1^ soil; ‘Li_2_’—100 mg Li_2_CO_3_ kg^−1^ soil; ‘S’—100 mM NaCl; ‘AA’—16 mM acetic acid; and ‘NO’—100 μM nitric oxide. Vertical bar diagrams represent the means of three independent replicates (n = 5). Vertical lines at the top of the bars indicate standard errors. Different letters in a graph (where three panels—‘Negative Control’, ‘Treatment: AA’, and ‘Treatment: NO’—together are considered as one graph) represent significant differences based on Tukey’s test at a 5% level of probability (*p* < 0.05).

GST activity was triggered under individual and combined stress treatments. Although NO non-significantly affected GST activity under various treatments, AA lowered the activity of GST significantly for AA+S+Li_1_ and AA+S+Li_2_ treatments (Figure 6A) compared to the stressed level. Compared with untreated control canola plants, the applied S and/or Li treatments, except S+Li_1,_ significantly reduced the PC content in canola leaves. The exogenous AA upregulated the PC content, whatever the stress applied, but increases of PC content for NO were nonsignificant compared to the respective stress conditions (Figure 6B). Compared with untreated control canola plants, MT content significantly decreased in S+Li_1_ and S+Li_2_ conditions, and there was no significant change for single stress conditions. The MT content improved in all the treatments under AA priming, but NO increased MT content significantly for only AA+Li_1_ and NO+Li_2,_ compared to their respective stress conditions (Figure 6C).

### 2.7. Effects of AA or NO on Endogenous NO, H_2_S, Cys, GABA, and SA Contents

**Figure 7:**
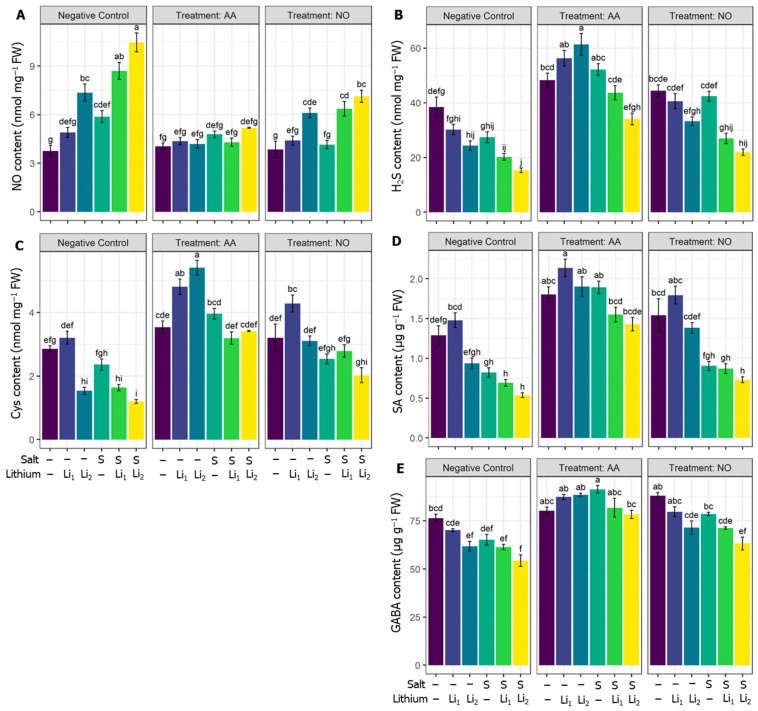
Effects of exogenous acetic acid (AA) or nitric oxide (NO) on (**A**) endogenous NO content, (**B**) hydrogen sulfide (H_2_S) content, (**C**) cysteine content, and (**D**) salicylic acid content, and (**E**) GABA content of canola seedlings grown under the presence or absence of different concentrations of lithium (Li) and/or salinity (S) stress. ‘C’—control (non-treated soil); ‘Li_1_’—50 mg Li_2_CO_3_ kg^−1^ soil; ‘Li_2_’—100 mg Li_2_CO_3_ kg^−1^ soil; ‘S’—100 mM NaCl; ‘AA’—16 mM acetic acid; and ‘NO’—100 μM nitric oxide. Vertical bar diagrams represent the means of three independent replicates (n = 5). Vertical lines at the top of the bars indicate standard errors. Different letters in a graph (where three panels—‘Negative Control’, ‘Treatment: AA’, and ‘Treatment: NO’—together are considered as one graph) represent significant differences based on Tukey’s test at a 5% level of probability (*p* < 0.05).

As illustrated in Figure 7A, single and co-exposure to Li and salinity significantly increased the endogenous content of NO in all the treatments except Li_1,_ compared to control plants. The exogenous AA significantly minimized endogenous NO whatever the treatment used, but not significantly for AA+Li_1_ and AA+S compared to their respective stress conditions. While NO treatment significantly reduced the NO content for NO+S+Li_1_ and NO+S+Li_2_, compared to their respective stress conditions (Figure 7A). The content of the signaling molecule H_2_S was adversely affected by Li_2_ and combined stressors compared with untreated control canola plants. The utilization of AA significantly upregulated H_2_S content irrespective of the treatment applied, compared to their respective stress conditions. Exogenous NO also curtailed the reduction in H_2_S content, which was significant for NO+S, compared to the respective stress conditions (Figure 7B).

Parallel to the reduction of H_2_S by the applied stressors, Cys content significantly attenuated under Li_2_, S+Li_1_, and S+Li_2_, compared with untreated control canola plants. AA or NO curtailed the reduction in Cys in all treatments compared to their respective stress conditions, but a significant effect of NO was noted under NO+S and NO+S+Li_2_ conditions compared to their respective stress conditions (Figure 7C). Regarding the hormonal status, SA content was significantly deregulated by S and/or Li, with the highest effect under S+Li_1_ and S+Li_2_, compared with untreated control canola plants. Effectively, AA ameliorated the reduction in SA content by single and combined stressors compared to NO application, which showed no significant effect (Figure 7D). The nonproteinogenic amino acid GABA was affected by saline/Li stress, but salinity escalated the damaging effect on GABA when cotreated with Li stress, compared with untreated control canola plants. In contrast, exogenous AA significantly increased GABA levels in all treatments compared to their respective stress conditions. Exogenous NO also increased GABA content, but this change was significant only for NO+S compared to its respective stress condition (Figure 7E).

### 2.8. Heat map and PCA Analyses

**Figure 8:**
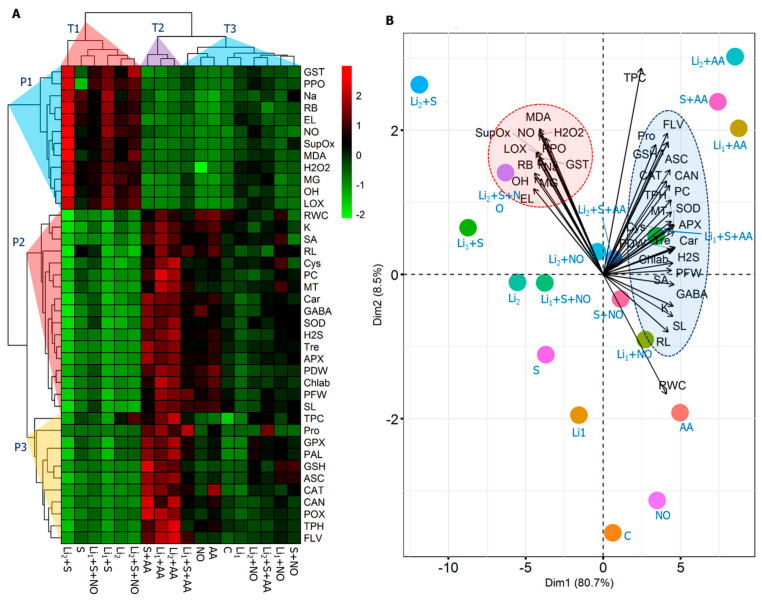
Heat map with Euclidean distance-based clustering (**A**) and principal component analyses (**B**) using all studied parameters. ‘C’—control (non-treated soil); ‘Li_1_’—50 mg Li_2_CO_3_ kg^−1^ soil; ‘Li_2_’—100 mg Li_2_CO_3_ kg^−1^ soil; ‘S’—100 mM NaCl; ‘AA’—16 mM acetic acid; and ‘NO’—100 μM nitric oxide.

Based on the hierarchical clustering, the collected data have three subcategories (cluster-P1, -P2, and -P3) (Figure 8A). Cluster-P1 is composed of GST, PPO, Na^+^, RB, EL, NO, Supox, H_2_O_2_, MDA, MG, ^•^OH, LOX, and RWC parameters. Relative to the untreated control, the cluster-P1 traits recorded an increasing trend due to the stresses applied. In contrast, those parameters were attenuated when stressed canola plants were treated with AA or NO (Figure 8A). K^+^, SA, RL, Cys, PC, MT, Car, GABA, SOD, H_2_S, Tre, APX, PDW, Chl *ab*, PFW, and SL variables were grouped in cluster-P2. Cluster-P3 is represented by TPC, Pro, GPX, PAL, GSH, ASC, CAT, CAN, POX, TPH, and FLV variables. Compared to the untreated control treatment, cluster-P2 and -P3 parameters exhibited a decreasing trend in stress-exposed plants; however, their values were increased in AA- or NO-primed stressed plants (Figure 8A). The PCA biplot shows the relationship between variables and treatments. Among the tested traits, 89.2% of the data variability was attributed to PC1 and PC2 (Figure 8B). Only the combined stress showed a moderate to strong association with PCA cluster-P1. On the other hand, AA or NO application to stressed plants was strongly linked to the traits of PCA in cluster-P2 and -P3 (Figure 8B).

## 3. Material and Methods

### 3.1. Growth Condition and Experimental Setup

This experiment was conducted using the canola cv. “*Sarw4*”. Initially, canola seeds were soaked in distilled water, sodium nitroprusside (100 µM), a source of NO, and acetic acid (AA) (16 mM) solution for 8 h in dark conditions. The concentrations of NO and AA were selected based on screening experiments, from which the concentrations that showed optimal growth were chosen. Afterward, the soaked seeds of each group were sown in pots filled with clay soil (2 kg) lined with plastic bags (10 seeds per pot). The pots were divided into six groups: (i) non-stress (control), (ii) lithium 1 (Li_1_, 50 mg Li_2_CO_3_ kg^−1^ soil), (iii) lithium 2 (Li_2_, 100 mg Li_2_CO_3_ kg^−1^ soil), (iv) salinity (S, 100 mM NaCl), (v) S+Li_1_, and (vi) S+Li_2_. All treatments were applied a week before seed sowing to allow interaction between the soil and the added solutions. For each treatment 5 pots were used, meaning each treatment had three independent replicates. The experiment was performed following a completely randomized design (CRD) model. The pots were irrigated with tap water to maintain the moisture content in the soil at around the field capacity. After 30 days of sowing, the plants were harvested and morphological and biochemical analyses were conducted.

### 3.2. Growth Criteria, Relative Water Content, and Pigment Content

Morphological attributes such as lengths of shoot (SL) and root (RL), plant fresh weight (PFW), and plant dry weight (PDW) of canola plants were measured following the methods described earlier [36,37]. For the estimation of the degree of root browning (DRB), a previously published protocol [38] was followed. The relative water content (RWC) of fresh leaves was analyzed based on a published method [39]. The RWC was calculated using the formula (fresh weight of leaves–dry weight of leaves)/(turgid weight of leaves (leaves placed in distilled water for 12 h)–dry weight of leaves). Chlorophyll (Chl) (Chl *a*, Chl *b*) and Car contents in leaves were evaluated by the Lichtenthaler and Wellburn [40] protocol. The fresh leaves were soaked overnight in ethyl alcohol (95%). The absorbances were recorded at 663, 644, and 452 nm wavelengths using a spectrophotometer.

### 3.3. Quantification of ROS, Electrolyte Leakage, Methylglyoxal, Lipoxygenase, and Lipid Peroxidation

Different ROS, such as H_2_O_2_ (hydrogen peroxide), O_2_**^•^**^−^ (superoxide anion), and ^•^OH (hydroxy radical), contents of canola plant leaves were estimated according to the proposed protocols [41,42,43]. For H_2_O_2_, a supernatant formed by macerating canola leaves in cold acetone was combined with a sulfuric acid–titanium dioxide reagent, where the absorbance of the produced color was measured at 420 nm. For O_2_**^•^**^−^ estimation, the supernatant of leaves extracted in PK buffer was incubated with a PK buffer and hydroxylamine hydrochloride mixture. To the last mixture, sulfanilamide and α-naphthylamine were added and the absorbance was recorded at 530 nm. For ^•^OH, the supernatant was mixed with KP buffer containing 2-deoxy-D-ribose in a water bath at 37 °C for 2 h. Then, boiling the last extract with glacial acetic acid, thiobarbituric acid, and NaOH for 10 min, the absorbance of the produced color was read at 532 nm.

Malondialdehyde (MDA) content [44], methylglyoxal (MG) content [45], and lipoxygenase (LOX) activity [46] in the leaves of canola plants were quantified according to the published protocols. Electrolyte leakage (EL) was determined following the method of Silveira et al. [47]. MDA content was assayed in the extract of leaves homogenized in trichloroacetic acid. After that, the extract was mixed with 2-thiobarbituric acid and then boiled for 30 min. After cooling, the absorbance was recorded at 532 nm. For MG, the leaves extract was incubated with 2,4-dinitrophenylhydrazine for 45 min at 45 °C and the absorbance of the developed color was measured at 432 nm. The KP buffer (pH 6) was used for extraction to determine lipoxygenase activity (LOX/EC.1.13.11.1). LOX activity was calculated following the rise in absorbance at 234 nm using an extinction coefficient of 25,000 M^−1^ cm^−1^. All the abosrbance readings were recorded using a spectrophotometer (Unico UV-2100 spectrophotometer, Missouri City, Texas, USA).

### 3.4. Measurement of Salicylic Acid, δ-Amino Butyric Acid, Proline, Trehalose, Na^+^, and K^+^ Contents

The foliar contents of salicylic acid (SA), δ-amino butyric acid (GABA), proline, and trehalose were analysed by following Warrier et al. [48], Zhang et al. [49], Bates et al. [50], and Li et al. [51], respectively. For SA, the leaves extract was mixed 0.1% ferric chloride. The absorbance of violet color was measured by spectrophotometry at 540 nm. For GABA, leaves were macerated in bi-distilled water, shaken for 30 min, and then filtered. Boil the mixture of filtrate, borate buffer, phenol, and sodium hypochlorite in the water bath for 10 min, and then cool in an ice bath. The produced blue color was monitored spectrophotometrically at 645 nm and GABA was used for the standard curve. A supernatant formed by grounding canola leaves in 5-sulfosalicylic acid was mixed with ninhydrin reagent and glacial acetic acid, and then heated in the water bath at 95 °C for 45 min. After cooling, toluene was added and shaken well with the last mixture. The absorbance of the organic layer was conducted at 520 nm. The supernatants of hot ethanolic extract of leaves were dried at 80 °C and then distilled water was used to produce trehalose extract. A mixture of the last extract and sulfuric acid was boiled for 10 min and then cooled on ice. Then, the last mixture and NaOH were cooled again, followed by adding anthrone reagent, and then heated for 10 min followed by cooling. The developed color was read at 630 nm using a standard curve of trehalose. For potassium (K^+^) and sodium (Na^+^) estimations, the dried grounded leaves (0.1 g) were digested as described by Tahjib-Ul-Arif et al. [52] and then estimated using a flame photometer (Carl-Zeiss DR LANGE M7D model, Jena, Germany) and applying the protocol of Williams and Twine [53].

### 3.5. Non-Enzymatic Antioxidants, Antioxidant Enzymes and Other Enzyme Activity Measurements

Ascorbic acid (ASA) [54], α-tocopherol (TPH) [55], and reduced glutathione (GSH) [56] on leaves were estimated by following the method reported earlier. The total phenolic compounds (TPC), anthocyanins (ACN), and flavonoids (FLV) were quantified based on the protocols of Kofalvi and Nassuth [57], Krizek et al. [58], and Zou et al. [59], respectively. In brief, ASA was determined by grounding leaves in trichloroacetic acid (TCA) (5%), followed by adding 10% TCA. Then, 10 times diluted Folin–Ciocalteu reagent was mixed and the intensity of the developed color was recorded at 760 nm. The TCA extract was used to evaluate GSH using DTNB, and the produced color was detected at 410 nm. α-tocopherol was detected in fresh leaves by usiing chloroform extract followed by using 2,2′-dipyridyl and ferric chloride reagents, and the red color produced was read at 520 nm. For anthocyanins, the mixture of canola leaves in acidified methanol was kept in a refrigrator for 5 h, then the supernatant was read at 550 nm. Phenolics were estimated by mixing methanolic extract with sodium carbonate and then Folin–Ciocalteu reagent, the color developed was read at 750 nm, and gallic acid was used as a standard. Total flavonoids were quantified in methanolic extract mixed with aluminum chloride, sodium hydroxide, and sodium nitrite, the developed color was recorded at 510 nm [41], and quercetin was applied as a standard.

The activities of antioxidant enzymes were analysed by following previously published protocols for superoxide dismutase (SOD/EC.1.15.1.1) [60], catalase (CAT/EC.1.11.1.6) [61], ascorbate peroxidase (APX/EC.1.11.1.11) [62], guaiacol peroxidase (POX/EC 1.11.1.7) [63], glutathione peroxidase (GPX/EC.1.11.1.9) [64], glutathione-S-transferase (GST/EC.2.5.1.18) [65], phenylalanine ammonia lyase (PAL/EC.4.3.1.5) [66], and polyphenol oxidase (PPO/EC.1.10.3.1) [63]. SOD was monitored in a medium of sodium carbonate buffer, KP extract of leaves, and epinephrine, and the autoxidation of epinephrine was recorded at 480 nm for 1 min. CAT was monitored in a reaction medium of enzyme extract, KP buffer, and H_2_O_2_ for 1 min and the reduction in the absorbance at 240 nm due to the breakdown of H_2_O_2_ was determined. APX activity was analyzed in canola enzyme extract in a reaction medium of H_2_O_2_, EDTA, KP buffer, and substrate ascorbate, and substrate oxidation was observed at 290 nm using an extinction coefficient of 2.8 mM^−1^ cm^−1^. POD activity was analyzed in a reaction medium of KP buffer, guaiacol, and H_2_O_2,_ and the absorbance was recorded for 1 min at 470 nm. GPX activity was quantified in KP buffer extract when mixed with KP buffer, GSH, Na_2_HPO_4_, and 5,5′-dithiobis-2-nitrobenzoic acid. Then, the changes were recorded at 412 nm w after 5 min. GST activity was screened in the enzyme extract of leaves in a reaction mixture of KP buffer, GSH, and 1-chloro-2,4-dinitrobenzene, and the changes were assessed for 3 min at 340 nm. PAL activity was tested by maintaining the enzyme extract with borate buffer and the substrate (phenylalanine) at 30 °C for 1 h, and then the reaction was ceased by introducing HCl. PPO activity was detected by incubating the enzyme extract at 25 °C with KP buffer and catechol for 5 min, and then diluted H_2_SO_4_ was added to stop the reaction. The formed purpurogallin was screened at 495 nm. All the abosrbance readings were recorded using a spectrophotometer (Unico UV-2100 spectrophotometer, Missouri City, TX, USA).

### 3.6. Determination of Nitric Oxide (NO) and Hydrogen Sulfide (H_2_S)

The protocols of Hu et al. [67] and Nashef et al. [68] were followed for NO and H_2_S using nitroblue tetrazolium and NaHS as standard curves, respectively. The supernatant of acetate buffer extracted leaves was combined with the re-extraction of the pellet. Charcoal and Greiss reagents were introduced to the clear supernatant and read at 540 nm. The H_2_S level was determined by macerating leaves in a KP buffer containing ethylenediaminetetraacetic acid. The supernatant was mixed with the extraction buffer and 5,5-dithiobis (2-nitrobenzoic acid) where the absorbance was monitored at 412 nm and a standard curve of NaHS was applied.

### 3.7. Determination of Phytochelatins, Cysteine, and Metallothioneins

Nahar et al. [69] estimated phytochelatins (PCs) by subtracting GSH, following Ellman’s method [56], from nonprotein thiols. The metallothioneins (MT) and cysteine (Cys) in the leaves were measured by the published protocols of Cataldo et al. [70] and Gaitonde [71], respectively. Cysteine was determined in a reaction mixture of leaf extract, NaHS, and acidic ninhydrin reagent. The nonprotein thiol of leaves was extracted by 5-sulfosalicylic acid and then their content was determined using Ellman’s reagent. PCs were calculated by subtracting nonprotein thiols and the previously determined GSH. The MT level was monitored by macerating the leaves in buffer (sucrose, Tris-HCl buffer, mercaptoethanol) then chilled ethanol and chloroform were introduced to the last supernatant and then again centrifuged. To the last supernatant, cold ethanol was added and then kept for 1 h at −20 °C. Another centrifugation was conducted to purify the metallothionein pallets, which were then washed with a mixture of ethanol, chloroform, and the buffer used for extraction. The pellets produced after centrifugations were then air dried and dissolved in a mixture of Tris-HCl and EDTA. To this dissolved MT, 5,5-dithiobis (nitrobenzoic acid) in phosphate buffer was added, and it was incubated at 25 °C for 30 min, then read at 412 nm.

### 3.8. Statistical Analysis

A two-way analysis of variance was conducted with the obtained data and then Tukey’s test at a 5% (*p* < 0.05) level of probability was applied. The hierarchical clustering (heat map) was performed using the package ‘pheatmap’ and principal component analysis (PCA) was performed using the packages ‘ggplot2’, ‘factoextra’, and ‘FactoMineR’ of R 4.1.2.

## 4. Discussion

Mitigation strategies for combined stressors differ from those for single stressors. Several studies have shown plant responses to salinity or Li stress and their mitigation using various exogenous chemicals, and the comparative effect of these two stressors alone and in combination requires further research. This experiment was intended to gain new insights into the responses of canola plants to individual and simultaneous salinity and Li stress and their mitigation using AA or NO. Lithium drastically impacts the morphogenetic traits of canola by impacting the condensation of DNA [7] and nucleic acid metabolism or modifying DNA conformation, thereby affecting the cell replication process and protein production [9]. Salinity restricts cell division and cell elongation (due to photosynthesis restriction), and CO_2_ assimilation, as well as stomatal closure [72], altering water relations and causing imbalances in the ionomics of plants [73]. In the present study, Li_1_ exhibited low toxicity to growth and developmental parameters (Figure 1 and Figure 2A–E), but its toxic effect became tangible when combined with salinity, and the situation became the worse under Li_2_+S compared to their corresponding single stressors Li_1_, Li_2_, or S (Figure 1A–E). This result indicates that the co-occurrence of the toxic effects of Li and salinity, Li_1_+S and Li_2_+S, intensifies the growth retardation of canola plants more than the individual stressors. Using AA or NO priming significantly alleviated the damaging effects of salinity and/or Li stress, which indicates stress- and elicitor-dependent responses. According to the heat map and PCA analyses (Figure 8), and the morphological view of the canola plants (Figure 1), the AA application showed better performance in reducing the effects of both individual and combined stressors than the NO application. This effect is associated with the upregulation of various physiological and biochemical responses, which will be discussed in detail, such as SA, H_2_S, oxidative homeostasis, K^+^ and Na^+^ equilibrium, photosynthesis, water status, osmoregulation, secondary metabolites, and antioxidant processes by AA application, which may be reflected in the phenotypic appearance and traits of canola plants under single or combined stressors.

The stress treatments induced the shortening of RL and the browning of roots, which were most obvious under the Li_1_+S and Li_2_+S treatments relative to Li or S stress. As a result, the canola seedlings suffered from low water access, as evidenced by lower RWC, especially under combined stress treatments (Figure 1F). It has been documented that high Li levels produce osmotic stress by altering the cytoplasmic structure of cells [9], while salinity causes osmotic stress by lowering the cells’ water potential. The reduction in RWC in stressed canola seedlings (Figure 1F) may cause physiological drought, which induces Chl breakdown via the PHEOPHORBIDE a OXYGENASE/phyllobilin pathway [74]. In support of this argument, we found that both RWC and Chl *a+b* content drastically decreased in both the Li_1_+S and Li_2_+S treatments (Figure 1F,G). However, the pretreatment of canola plants with AA or NO inhibited the reduction of RWC and Chl (Figure 1), which might be a sign of an increase in the photosynthetic rate [73]. Plants adjust osmotic stress by synthesizing various compatible solutes, such as soluble proteins, soluble sugars, free amino acids, glycine betaine, Pro, and trehalose [75]. AA and to a lesser extent NO increased the photosynthetic pigments, thereby stimulating the production of various organic components used by cells as osmolytes. In the present study, AA significantly enhanced proline content, which may confer high resistance to stress because a high level of proline in cells during stress conditions shields cells and prevents toxicity from damaging the plant tissues [76]. Furthermore, AA and NO priming enhanced trehalose content, especially under single stressors, but AA significantly enhanced trehalose and RWC under the combined stressors. Trehalose acts as an energy sink, signaling, and antioxidant molecule, and it potentially protects the enzymes, proteins, and lipid membranes from dehydration, as well as saving the biological organs from the deterioration induced by harsh conditions [77].

Ogawa et al. [23] reported that the application of AA to rice seedlings produces GABA in roots by inducing signals in the roots, and then translocate the GABA to shoots. Heat map analysis indicates a positive correlation between AA and the promotion of GABA content under stress conditions, while NO only has a positive effect under single stressors. Similarly, NO enhances GABA biosynthesis in bamboo plants exposed to low temperatures [78]. Kaspal et al. [79] reported that a high content of GABA under abiotic stress may directly or indirectly upregulate other transport proteins or channels (e.g., aquaporins) that confer abiotic stress tolerance. GABA has been found to negatively regulate aluminum-activated malate transporters, which can upregulate the Na^+^ level [80,81,82] by enhancing cytosolic K^+^ retention and excluding Na^+^ [83]. Therefore, the modulation of GABA through AA or NO application may promote ion homeostasis and enhance stress tolerance [84]. The application of NO [22] and AA [26] has been reported to upregulate plant nutrient uptake and leaf photosynthesis, leading to improved growth traits under salinity stress.

In the present study, only Li stress did not increase Na^+^ content, while salinity stress elevated Na^+^ accumulation, particularly when coexisting with Li. Excessive Na^+^ uptake led to reduced water uptake (Figure 1F) and K^+^ absorption (Figure 5A,C), indicating that ionic stress was causing tissue dehydration, resulting in growth stunting and chlorosis (Figure 1 and Figure 2). Moreover, excessive Na^+^ uptake can damage cellular structures such as Chl, accounting for impaired photosynthetic machinery and imbalances in various metabolic activities [85,86]. However, plants supplied with AA or NO were helped to limit the uptake of sodium ions, especially under the combined stressors. Similarly, Zhang et al. [87] reported the re-establishment of K^+^ and Na^+^ homeostasis in perennial ryegrass through AA, which also elevated ROS quenching capacity, and modulated stress-protection hormone biosynthesis and signaling pathways, supporting the finding of the present experiment. Our study consistently revealed a significant reduction in K^+^ content and a marked increase in Na^+^ range under stress treatments (Figure 5C,D). Interestingly, a robust imbalance in ionic homeostasis was noted in the Li_1_+S and Li_2_+S treatments (Figure 5C,D). In this regard, salinity induces an ionic homeostasis imbalance, with K^+^ uptake being antagonistic to Na^+^, while, Li negatively affects K^+^ uptake by sharing the same K^+^ transport carrier across membranes [9], accounting for the depletion of K^+^ content in canola leaves. The co-occurrence of Li and salinity stress further weakens K^+^ content and physiological reactions related to K^+^ relative to their corresponding single stressors. AA and NO efficiently contribute to balancing the antagonistic effect of Na^+^ on K^+^ uptake, increasing its accumulation. AA treatment benefits canola plants in terms of stomatal conductance, chlorophyll and protein biosynthesis, enzyme activation, protection of membrane structure, and reduced activation of stress hormones [26,88], while NO enhances Na^+^/K^+^ balance by triggering the activities of the proton-pumping ATPase, H^+^-pumping inorganic pyrophosphatase [89], and Na^+^/H^+^ antiporters, as well as potassium channels [90].

Both single Li and salinity stressors trigger the accumulation of ROS, which is supported by the findings of this study. The combined stressors (Li_1_+S and Li_2_+S treatments) induce oxidative stress, resulting in the production of higher levels of H_2_O_2_, O_2_^•^, and ^•^OH compared to the control and single stressors. This excessive ROS and membrane dysfunctions can disrupt the enzymes involved in chlorophyll synthesis and assembly, affecting their functionality [91]. The single stressors and combined stressors impact plant metabolism at the membrane level, as indicated by increased levels of EL, lipid peroxidation (MDA), LOX activity, and MG content in the leaves of canola plants (Figure 1H and Figure 2). Plants possess antioxidant systems that help to reduce oxidative stress [92]. In our study, the applied stresses were severe enough that the defending antioxidant system failed (Figure 4 and Figure 5) to shield against the rising levels of ROS (Figure 2A–C), which ultimately deteriorated the membranes (Figure 2D–F). Importantly the generation of ROS, membrane damage, and imbalance in antioxidant systems were more prominent under combined stressors rather than individual stressor conditions (Li_1_, Li_2_, and S). In this sense, the intensified oxidation processes observed, especially under combined stressors, could inhibit antioxidant enzymes or deregulate the transcription of genes involved in antioxidant production [9]. This could be the most striking reason for the impaired plant growth and developmental disruption in the Li_1_+S and Li_2_+S treatments (Figure 1, Figure 2, Figure 3, Figure 4 and Figure 5). This hypothesis was also validated via the heat map generation and PCA analysis of our data (Figure 8).

The priming with AA or NO reduces ROS accumulation and detoxifies membrane damage, with AA being more effective than NO in the attenuation of oxidative stress. In this regard, AA mediates lipid metabolism under stress conditions, accumulating the lipids needed for the membrane bilayers, protecting membranes from dysfunction [93], and stimulating the antioxidant capacity of salt-stressed plants as a defense strategy to withstand ROS accumulation [19]. On the other hand, NO mitigates the oxidative burst by adjusting ROS levels, improving cell stability and elasticity by strengthening the phospholipid bilayer and improving the fluidity of the membrane [94]. NO also triggers antioxidant enzyme activity and reduces the uptake of heavy metals by plants [95,96]. Additionally, NO restricts the oxidative burst in abiotic-stressed plants by capturing O_2_**^•^**^−^ and lipid radicals and promoting antioxidant activity [97,98]. Therefore, it can be inferred that AA or NO modulate osmotic adjustment and endogenous GABA levels, restore Na^+^ and K^+^ balance, and trigger antioxidant systems, ultimately quenching ROS levels, maintaining cell membrane stability, and enhancing canola tolerance to Li and/or salinity stress.

In the present study, the applied stresses activate the generation of ROS and reactive nitrogen species (RNS). Li and salinity stress caused an imbalanced accumulation of endogenous NO. We found that the combined stressors adversely disrupted growth and many biochemical processes in canola seedlings, impacting nitrosative effects. This reaction may destroy various physiological pathways in plant cells, such as the nitration process which negatively affects proteins, nucleic acids, and lipids, causing nitrosative stress [99]. This indicated that the Li_2_, salinity, and, more prominently, the combined stressors, increase the susceptibility to both nitrosative and oxidative damage. Pre-treating the plants with AA or NO upregulated the endogenous NO content, but excessive NO accumulation is controlled by NO acting as an endogenous signaling molecule. Such a strategy may be valid for single stressors, exerting a beneficial effect on the canola plants, but not for the combined ones. While AA was highly efficient in halting the endogenous NO, whatever the stress imposed, compared to their corresponding levels under the stressors. A similar role of NO application in stress tolerance was mediated by enhancing the scavenging RNS and ROS, stimulating the production of osmoprotectants, increasing antioxidant activities/molecules, and upregulating gene expression [100].

In the present investigation, the phenolic compounds significantly increased under a single stressor. This accumulation of phenolic compounds during harsh conditions can act as a signal that initiates a cascade of pathways, eventually accounting for elevated stress responses [101]. However, compound stress resulted in substantial reductions in carotenoids, flavonoids, and anthocyanin contents. This indicates the failure of secondary metabolites to aid in withstanding the imposed stress, necessitating the support of various stimulators to reinforce plant tolerance and development under complex stress. This effect was concomitant with a decrease in an important secondary metabolite producing enzyme, phenylalanine ammonia lyase. Interestingly, the mitigation effect of the used elicitors is produced by triggering the activity of PAL. Thus, the aforementioned secondary metabolites were promoted by AA or NO under single and co-stresses, which aided in conferring tolerance to the canola plants.

Salicylic acid, a simple phenolic compound and an important plant hormone synthesized by PAL [102], was also included in the study. As a plant stress hormone, SA is regarded as an active signaling molecule implicated in stress responses [103]. In the present work, the co-occurrence of salinity and lithium negatively impacted the content of SA compared to single-stress and control plants, revealing hormonal imbalances under combined stressors. Consequently, the regulatory roles of SA for various physio-biochemical attributes of canola plants were downregulated under these conditions. It is evident that AA or NO enhanced the production of SA under single or combined stressors. In this regard, SA induces stress tolerance and enhances plant phenotypic traits by increasing chlorophyll content and antioxidant capacity, ultimately promoting the performance of the photosynthetic system and lowering oxidative stress [104]. The role of AA in increasing SA under salinity stress as a tolerance mechanism has been reported in strawberry plants [26], and the interaction between NO and SA can act as a modulator of multifaceted plant processes and mitigate the damaging impacts of stress conditions [28]. Such interactions govern plant development, with SA controlling NO and its related molecules, which then upregulate SA levels, thus decreasing NO modulation of oxidative bursts [105].

Hydrogen sulfide is an active signaling compound and is implicated in the induction of plant stress responses. The single or combined stressors reduced endogenous H_2_S levels relative to the control, revealing the susceptibility of canola plants to the imposed stresses. On the other hand, the results of PCA levels indicated stronger positive associations between NO- or AA-primed stressed plants and the content of this signaling molecule than those observed under stress conditions. Numerous studies have confirmed the functional parallelism of NO and H_2_S in mediating multiple physiological pathways under harsh conditions [106]. Many investigations have deduced the involvement of H_2_S in NO-mediated salinity or heavy metal stresses of many plants [107,108,109], which supports the role of NO, or possibly AA, in the mitigation of salinity and/or Li stress through the upregulation of endogenous H_2_S.

Sulfur metabolic products are key defense pathways in the plant cell against abiotic stress. PCs, MTs, and GSH are key feature defense components, which stimulate the sequestration of heavy metals in vacuoles. GSH is a multifunctional metabolite that helps to modify sulfur assimilation and the storage and transport of its reduced forms, regulates cell differentiation and cell death, maintains the redox status, quenches the reactive ketoaldehydes (MG), which are a precursor of PCs, and detoxifies the xenobiotics, thereby alleviating biotic and abiotic stresses [110]. In addition, MT shares with GSH in the duties of sequestration and chelation of heavy metals. Although salinity stress and Li_1_, herein, had no significant effect on various chelating agents, the presence of Li, even at the lowest concentration, deregulated the chelating factors GSH and PC, which attenuated the sequestration of toxic Li, hence preventing its ability to hinder the metallic toxicity under combined stress. In connection, a rate-limiting factor for GSH biosynthesis, cysteine, was also negatively affected by Li_2_ and combined stress, which supported the aforementioned reduction in GSH, PCs, and MTs. Hence, the production of sulfur-related compounds, with their multidefensive roles, were impaired when Li was at a toxic level and when jointly interacting with salinity. MTs are metal chelators and ROS-scavenging compounds rich in cysteine [111]. In the present study, MTs were also reduced under combined stress, thus their role in chelation or detoxification of ROS levels was limited, revealing the failure of major metal chelation and detoxification mechanisms which negatively affect plant growth and metabolism. These results showed that the harmony of plant responses to individual and combined stresses is a complex matter and more studies at the genetic level must be addressed. AA or NO potentially stimulated cysteine production whatever the treatment used, thus, the whole sulfur pool was sufficiently stimulated to cope with the toxic Li_2_ or combined stressors. It has been reported that the interaction of GSH and GSTs modifies cellular detoxification by a wide variety of electrophilic compounds in cells because of their affinity to deregulated xenobiotics [112]. Unlike the other GSH-related compounds, the activity of GST increased highly significantly for Li_2_ and the combined stressors. Also, PCA and heat map analyses confirmed its positive association with imposed stress. Such activation of GST could be a specific response to the applied stressors as an indication of the presence of xenobiotics that adversely affect canola. Thus, the application of AA or NO retarded its accumulation under stress conditions where the consequences of single- or bi-stressors induced the attenuation of GST activity. Similarly, PPO also exhibited a positive correlation with combined stress, suggesting that a high level of PPO may degrade pigments and induce the early senescence of canola plants. Thus, AA or NO applications, as anti-stress compounds, reduced its activity as a sign of the stress amelioration effect of both protectants.

In general, NO had a slight protectant effect on the combined stressors relative to single stressors, and the magnitude of regulatory effects was much higher in AA-treated plants relative to NO, revealing the different responses of the canola plant to the protectants used. Although there is a high response to the single stressor of the AA- or NO-treated plants, the influence exhibited a specific response under combined stressors. This indicated a certain degree of independence between the regulatory mechanisms of plant responses to single and combined stressors. According to the heat map and PCA analysis (Figure 8), and a morphophysiological perspective on the canola plants (Figure 1), the superior performance of AA was noticed at several checkpoints, such as (1) exogenous AA reduced ROS contents highly significantly compared to NO application (Figure 2A–C); (2) exogenous AA application showed a markedly greater increase in nonenzymatic antioxidants, such as ASC, GSH, TPH, ACN, TPC, FLV and Car, than NO application (Figure 3); (3) exogenous AA application showed a more robust increase in SOD, CAT, POX, APX, and GPX activity than NO application (Figure 5A–E); (4) exogenous AA significantly increased the osmolytes, such as trehalose and proline, content, which subsequently increased the RWC in plants (Figure 5A,B); (5) exogenous AA regulated some of the plant hormones and signaling molecules, such as SA and H_2_S, and other important regulators, such as Cys, endogenous NO content, PC content, MT content, and PAL activity, more than exogenous NO application under Li+S stress conditions (Figure 7B–D and Figure 8). Overall, these finding suggest that AA is a potential plant growth regulator in combined conditions of salt and Li stress. Moreover, the PCA and heatmap of all the studied parameters revealed that the Li+S stress response works differently than the response to individual salinity or Li stressors (Figure 8). A strong and positive correlation was found between combined Li+S stressed treatments, ROS content, and lipid peroxidation in canola plants (Figure 8). These results indicate that combined stress caused more oxidative and nitrosative damage, resulting in more membrane breakdown, which caused stunted plant growth. However, PCA showed a strong and positive association between nonenzymatic antioxidants, enzymatic antioxidants and AA treatment (Figure 8). These results indicate that the antioxidant system is a key area in withstanding combined stressor-induced oxidative bursts.

## 5. Conclusions

Our study provides compelling evidence that combined stress treatment with Li and salt is more phytotoxic, which inhibits plant growth and biomass by damaging several physiological and biochemical mechanisms, including (i) reducing RWC and disrupting osmoregulation, (ii) destroying photosynthetic pigments, (iii) accumulation of toxic Na^+^, (iv) enhancing oxidative stress via ROS, MG, MDA, and lipoxygenase accumulation, (v) destabilizing the antioxidant capacity, and (vi) unbalancing phytohormones and plant stress regulators. However, AA showed superior performance in counterbalancing the combined stress-induced damage. In light of these results, it can be inferred that AA application could be a viable and cost-effective strategy to fight against salt and Li combined stress. However, further studies at the field level should be conducted to establish the role of AA in salt and Li combined stress.

## Data Availability

The datasets generated during and/or analyzed during the current study are available from the corresponding author on reasonable request.
